# Measuring restoration quality in urban forest greenways: insights for planning and management

**DOI:** 10.3389/fpsyg.2025.1596154

**Published:** 2025-07-18

**Authors:** Hong Wang, Yuxiang Liu, Ming-Juan Zhang, Xinxin Wang

**Affiliations:** ^1^Nanjing Institute of Landscape and Forestry Science, Nanjing, China; ^2^Department of Landscape Architecture, College of Horticulture, Nanjing Agricultural University, Nanjing, China

**Keywords:** green space, urban forest, route, attentional level, health benefits

## Abstract

**Introduction:**

Greenways play a crucial role in enhancing citizens’ quality of life by providing restorative environments, particularly in settings such urban forests. While existing research underscores the superior restorative qualities of natural settings compared to urban environments, our understanding of how various urban forest greenways and seasonal variations shape restorative experiences remains limited.

**Methods:**

This study investigates the restoration effects of various greenway scenes within the urban forests, including one urban greenway, one wilderness greenway and one tended greenway. A total of 55 university students viewed six videotaped greenway scenes in a randomized order. The urban and wilderness greenways were presented only for the summer scenes, while the tended greenway was shown across all four seasons. Physiological responses were measured using Electroencephalography (EEG), while psychological responses, including attentional levels and restorative experiences, were assessed with the Necker Cube and the Perceived Restorativeness Scale (PRS) respectively.

**Results:**

This study confirmed previous research that the natural environment restored attention, and watching a combination of different types of greenways and seasons increased attentional level. Findings suggest that tended greenways offer more effective restoration compared to urban greenways. Notably, tended greenways in spring, summer, and autumn were more restorative than winter scenes.

**Discussion:**

These findings highlight how different types of greenways and seasonal variations can affect perceived restorativeness. They provide valuable insights for the planning and management of urban forest greenways, emphasizing the importance of considering route selection, planting design, and maintenance to enhance restorative benefits.

## 1 Introduction

Urban citizens need resources such as nature contact to restore their depleted attention and reduce the stress of daily life (Collado et al., 2017; van den Berg et al., 2007), and promote better health outcomes (Berman and Krpan, 2014; Hartig et al., 2011). Although numerous studies have demonstrated that natural environments offer multiple benefits (Soga et al., 2021; Twohig-Bennett and Jones, 2018), and can generally provide better restorative effects than the urban settings (Menardo et al., 2021), we should consider nature and the urban environment as existing on overlaid spectrums (Hartig et al., 1997), rather than as two extremes. Urban forest greenways, typically integrated into the built environment, offer accessible contact with nature, and can provide restorative effects for those who visit them (Chang et al., 2020; Tyrväinen et al., 2014; Wang et al., 2023; Xie et al., 2022). Even within urban forest greenways, we can distinguish different subtypes based on the spectrum. Evaluating the restorative quality of these different settings is important, as natural settings may consist of diverse elements. The amount, type and arrangement of vegetation and other materials can significantly influence the restorative quality of these settings (Probst et al., 2024; Wyles et al., 2019). We should move beyond the natural-built dichotomy in research on restorative environments and focus on how to enhance existing settings to create more restorative environments.

Both Stress Recovery Theory (SRT) and Attention Restoration Theory (ART) offer valuable insights into the process of restoration. SRT emphasizes the importance of distancing from stress and engaging with unthreatening natural stimuli, which can be reflected in both physiological and affect responses (Ulrich, 1983). ART focuses on attention restoration needs and cognitive aspects, proposing that restorative environments promote recovery by providing four key characteristics: “being away,” “soft fascination,” “extent,” and “compatibility” (Kaplan, 1995). In the context of urban forest greenways, its landscape elements and structural arrangement may influence these characteristics, and thereby impacting users’ restorative experience.

When distinguishing different subtypes of landscape composition within a landscape type, previous research generally follows two primary approaches: One categorizes based on the elements observed in the scene, referring to the distinct hardscape or natural components that make up a landscape. The other emphasizes the structure of the arrangement, which may include route selection and spatial configuration created by those components, particularly the plantings on both sides of the trail. For the first direction, studies often using photos as the stimuli, and have demonstrated that even a short time exposure to nature can restore attention and relax tired minds (Lee et al., 2015; Yao et al., 2021). Several studies have shown that the restorative potential of landscapes can be predicted by the visible components of the scene, such as grass, trees, bushes, water bodies, and flowers (Deng et al., 2020; Huang et al., 2020; Kuper, 2017; Wang et al., 2019), while fewer buildings enhance their attractiveness and reduce mental stress, as demonstrated through objective measurements, conjoint methodology, and data mining techniques (Nordh et al., 2009; Nordh et al., 2011; Jahani and Saffariha, 2020). These findings indicate that natural settings, characterized by living plants and other natural features, can support greater restoration, as they facilitate experiences of “being away” and “soft fascination.” However, many studies in this area rely on static images to isolate individual elements. While this approach is effective for examining the impact of visual components in single scenes or small urban park environments, it falls short when exploring larger areas or dynamic, path-oriented experiences. Urban forest greenways, being linear spaces, often following natural features and including trails for walking and other outdoor activities (Li et al., 2024; Parton, 2023; Wang et al., 2022). It is necessary to adopt dynamic presentations based on walking experience, to evaluate the impact of element compositions of urban forest greenways on psychological restoration.

For the second direction, the structural arrangement of landscapes, which may relate to both greenway routing and plants arrangement, could affect the restorative potential of urban forest greenways. Some greenways within the urban forests are situated adjacent to major traffic corridors, either at the edges of the urban forest park, or near the roads that pass through the park. Due to the alignment of these greenways being close to roads, they give a sense of proximity to the urban environment (urban greenways). Other urban forest greenways are positioned away from these roads, creating more enclosed, evenly distributed green spaces along the trail. Choosing routes away from noise and traffic may enhance the sense of “being away,” making it easier for individuals to disconnect from daily life and enter a state of relaxation. Planting strategies can also vary widely, and different vegetation types and planting communities may also impact restorative effects (Duan et al., 2024; Peng et al., 2025). From the management perspective, greenways can be less maintained, preserving a more natural state (wilderness greenways), or can be carefully designed and managed, with visible signs of maintenance (tended greenways). These varying subtypes of urban forest greenways may lead to different spatial structures and quality, which in turn can affect people’s restorative experience. Although research specifically on urban forest greenways is still relatively limited, there is a body of studies on urban greenways in general (Xie et al., 2022), highlighting the role of different types of vegetation in psychological restoration (Cao et al., 2024; Jiang et al., 2024). Studies examining the relationship between biodiversity and restorative potential have yielded mixed results (Houlden et al., 2021). Evidence has shown wilderness, while boosting biodiversity, may evoke insecurity and be perceived as less restorative (Beute et al., 2023; van den Berg and Konijnendijk, 2018), whereas tended landscapes with visible signs of maintenance may support stronger restorative experience (Hartig, 2021), and provide greater compatibility. In research on urban forest greenways, the types and qualities have seldom received attention (Han et al., (2021)), with only a few studies discussing the differences between greenways in the urban and rural areas, and their various locations adjacent to different natural resources (Cao et al., 2024). The structural arrangement of urban forest greenways deserves further investigation, particularly given the potential for feasible improvements in planning, design, and management, along with the need to balance design methodologies with management costs to create a more restorative environment.

When considering both the elements and structure in natural scenes, the seasonality of the environment must not be overlooked. Previous studies have generally focused on late spring to summer conditions or used summer photographs, which showcase lush vegetation, highlighting the characteristics of nature during its peak growing season. The advantage of this approach is that it provides insights into the characteristics of plants during their most vibrant period. However, not every area in the global can experience lush vegetation across the year, some regions experience prolonged snow cover, while others undergo distinct seasonal changes. It is therefore meaningful to explore seasonal variations, as different seasons can not only alter the perceived greenery amount and color of natural elements but may also change vegetation structure (e.g., through the loss of leaves in winter). Seasonal changes can alter the setting of soft fascination, as different color stimuli and those colors evolve throughout the year. Among the limited existing literature in this field, studies in high-latitude areas of China and Canada have shown that winter landscapes with snow-covered terrain offer restorative benefits like those of summer landscapes (Brooks et al., 2017; Zhu et al., 2023). In contrast, a study in the U.S. found that models featuring foliage (both foliated and evergreen) were preferred and rated higher for restorative potential than defoliated models, emphasizing the importance of plant types in seasonal landscape design (Kuper, 2020). Current research on seasonal differences in landscape restorative potential is inconsistent: Some studies find spring most effective for stress relief, while others highlight the benefits of autumn foliage (Bao et al., 2023; Chen et al., 2023; Paddle and Gilliland, 2016; Xiang et al., 2021; Yin et al., 2024), highlighting the need for further investigation.

Given these considerations, when exploring urban forest greenways, it is essential to consider not only the structural differences caused by element variation but also the seasonal changes. Seasonality’s influence is particularly important in subtropical and tropical regions with distinct seasonal variations, as it can guide plant configuration and design. Considering seasonal differences serves two main purposes: First, it helps us understand that the restorative benefits of the same location may vary across different seasons. This suggests that the restorative potential of natural settings might change cyclically over time, with some seasons being more effective in restoring attention or shortening recovery time. Second, exploring the restorative differences of scenes with the same vegetation structure but across different seasons can inform plant selection. Taking seasonality into account highlights the potential restorative differences between deciduous, evergreen, and colorful-leaf plants.

This study aims to measure and compare the restorative effects of different subtypes of urban forest greenways, including tended, urban, and wilderness greenways, in exploring how the elements and structures of landscapes may influence restoration. The measurements utilized both physiological and psychological tests to assess participants’ responses to videos of different types of urban forest greenways. The study also investigates the restorative potential of greenways across four seasons, with a specific focus on the well-designed tended greenway as an example. The goal is to reveal differences in restorative potential linked to seasonal landscape experiences and to provide valuable insights for greenway planning, management, and plant selection.

We hypothesized as follows:

*H1*: Watching a dynamic walking video featuring a blend of scenes and seasons of urban forest greenways can effectively alleviate subjects’ attention fatigue and enhance their cognitive performance, as evidenced by a reduced frequency of flips in the Necker Cube before and after the video-watching session.

*H2*: For the same season with abundant tree cover (summer), different subtypes of greenway landscapes within urban forests, such as tended greenways, urban greenways, and wilderness greenways, may provide varying restorative effects. Specifically, compared to urban greenways, tended greenways are expected to be more effective in promoting restorative benefits, resulting in higher alpha wave values.

*H3*: For tended greenways across four seasons, we anticipate seasonal differences, with scenes featuring more green or colorful foliage providing greater restoration benefits than leafless winter season, leading to higher alpha wave values in spring, summer, and autumn scenes.

*H4*: Further formalizing the above hypotheses using psychological indices, we anticipate that tended greenways can provide a better restorative experience than urban greenways, leading to higher values in perceived restorativeness (H4a). Additionally, seasons with more greenery and colorful foliage are expected to offer a better restorative experience than the leafless winter season, resulting in higher values in perceived restorativeness (H4b).

It is an open question whether the wilderness greenway will produce more beneficial restoration than that of the urban greenway, as they can offer more greenery and natural views. In addition, although we did not make specific hypotheses about the restorative differences among the spring, summer, and autumn seasons of tended greenways, we are interested in this aspect, as these scenes, despite being based on the same planting structure, may offer varying levels of restoration due to differences in visual qualities and colors.

## 2 Materials and methods

### 2.1 The environments

The greenway scenes were selected from two urban forest mountains in Nanjing city, Jiangsu Province, China. Nanjing sits on the banks of the Yangtze River, covering an area of 6,587 km^2^, with a permanent population of 9.5 million by the end of 2023 (Bureau of Statistics of Nanjing., 2024). Nanjing’s annual temperature was 16.9°C and annual precipitation was 819.8 mm in 2022 (Nanjing Municipal People’s Government, 2023). It has a humid subtropical climate and four distinct seasons, and the vegetation types include coniferous forest, deciduous broad-leaved forest, mixed deciduous and evergreen broad-leaved forest, shrubs and grass. The leaves of some trees change color in response to the changes in temperature and sunlight throughout the year, formulating different seasonal scenes in the city.

Two mountains in Nanjing were selected as sites for the greenway scene. One of them is the Purple Mountain Forest Park (32◦3′ 44″N, 118◦51′ 11″E), which is located in the central city area of Xuanwu District, with an area of approximately 30.1 Km^2^ and a forest coverage rate of 76.8%. The greenway around the Purple Mountain is nearly 30 km long, connecting many places of interest in the mountain, as well as public transportation. Most of the trees along the Purple Mountain greenway are planted by humans and are regularly maintained. The other mountain selected is Lao Mountain (32◦6′ 6″N, 118◦36′ 6″E), which is located in the Jiangbei New District, with an area of approximately 50.6 km^2^ and a forest coverage rate of over 90%. The greenway around the Lao Mountain is about 30 km long, some of which are cycling routes built to host the Youth Olympic Games, while others are used to connect the attractions and transport stops in the nearby.

In the initial investigation, the authors conducted a field survey, and the second author took 764 pieces of photos of the Purple Mountain and 626 pieces of photos of the Lao Mountain. The authors analyzed the landscape components of the photos through pixel segmentation and clustering analysis (not reported in this paper) and the surrounding environment of the greenway sections, and then identified the subtypes of greenways around the mountains. We also consulted with the forest managers and visitors for both mountains to determine whether the classifications were in line with their perception and experience. Based on these procedures, the three subtypes of urban forest greenways identified are the tended greenway, the urban greenway, and the wilderness greenway. As actual urban forest greenway scenes, they incorporate interconnected factors such as landscape elements, route selection, and plant arrangement in a more pragmatic way. The tended greenway is characterized by well-maintained planting elements on both sides, and pass through the urban forest and connecting various attractions within the mountain in terms of route selection, creating a parkway-like scene. While the urban greenway is also a subtype of the urban forest greenway system, it integrates soft natural landscapes and hardscapes. These greenways typically run parallel to city roads and connects transport stations within the park and in the surrounding areas, highlighting a different route selection strategy compared to tended greenway. The wilderness greenway, similar to the tended greenway, runs through urban forests and has plantings on both sides. However, it preserves a more natural landscape with minimal human intervention, retaining a large amount of naturally growing flora, thus maintaining the wild and natural character of the environment, while also promoting an economical landscape management strategy. We used real greenway videos of the subtypes of urban forest greenways to capture the holistic and interconnected factors that shape them, providing a more realistic and immersive experience for the viewers.

The study selected filming locations for each subtype of the urban forest greenway: The tended greenway nearby Youju Road in Purple Mountain, the urban greenway along Huanling Road in Purple Mountain, and the wilderness greenway along Zhenqi Road in Lao Mountain. Both the tended and urban greenways were selected from Purple Mountain, as it is centrally located, with more management compared to the newer district area of Lao Mountain, from which the wilderness greenway was selected. The particular sections of the representative greenways were selected based on the photos taken by the second author. We selected longer continuous sections for each greenway subtype to allow for uninterrupted video recording and consistency. Factors such as fewer intersections and less visitor congestion were also considered to minimize disruptions during video recording.

Six videos were recorded as the stimulus, including four seasonal scenes (spring, summer, autumn, and winter) of the tended greenway, as well as summer scenes of the urban and wilderness greenways (Table 1). The tended greenway was selected for seasonal analysis due to its significant planning and maintenance requirements in urban forest management. Investigating its year-round dynamics allows for an assessment of how vegetation conditions influence restorative effects across different seasons. Videos were recorded using a camera and gimbal on clear days from January 2021 to January 2022. The camera was positioned at an eye level of 1.60 meters, with the photographer walking at a slow pace (2.8 km/h) (Wang et al., 2021) down the center of the greenway to simulate the visual experience of a pedestrian. An audio recorder (Sony ICD-PX470) was used to capture audio along the greenways to ensure good sound quality and avoid equipment noise from the camera.

**TABLE 1:** Screenshots from edited videos of three subtypes of urban forest greenways, including one same tended greenway showcasing the four seasons.

Subtypes of urban forest greenway	Screenshots from the videos
**Tended greenway**	
(Spring) 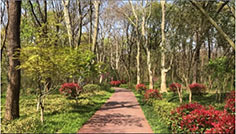	(Summer) 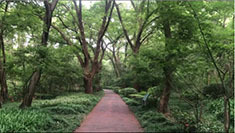
(Autumn) 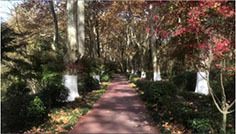	(Winter) 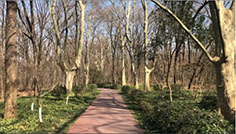
**Urban greenway**	
(Summer) 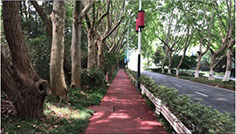	
**Wilderness greenway**	
(Summer) 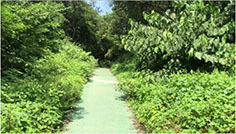	

After collecting the footage, editing was performed to remove segments containing visible pedestrians or other distracting elements. Specifically, video segments where pedestrians were located within the lower two-thirds of the frame (closer to the viewer along the pathway) were removed, as the actions of pedestrians could distract the viewer and lead to biased judgments, which would not align with the objectives of this experiment. For the same reason, any footage containing textual signs that could cause misinterpretation or confusion, such as wildlife warning signs, was also removed if they appeared in this area. Because some pathways have specific signs while others lack such uniform management, we wanted to avoid the potential influence of these signs on the viewer’s assessment.

After modifying the footage, the original sound was removed and replaced with audio recorded using the recorder. The audio used for each greenway type video was captured from the original sound of that specific greenway. The audio was then adjusted by carefully removing any parts with mismatched noise, such as clear speech and noticeable footsteps (e.g., someone running by), to better match the video content. This process resulted in the creation of 30-s experimental video samples representing each greenway setting. In the videos, we did not provide information about the filming locations, only presenting the greenway scenes. Using a 30-s video allows for recording brainwave activity in a resting state (Thompson et al., 1992) while also reducing viewer fatigue from prolonged exposure to the same type of scenes.

### 2.2 The participants

The participants in the experiment were all university students. Given that they experience high-intensity studying on a daily basis, attention restoration and alleviating fatigue are particularly important for them. The project was sponsored by the Department of Landscape Architecture, College of Horticulture, Nanjing Agricultural University, and the study protocol was approved by the Experimental Animal Welfare and Ethics Committee of Nanjing Agricultural University, and all the participants provided written informed consent. A social network discussion group was established to reach the potential participants. The recruitment criteria included that the participants should be non-design major university students, aged between 20 and 25, right-handed (i.e., using the right hand for eating and writing), with normal corrected visual acuity and no diagnosed cardiovascular diseases. Non-design major students were specifically selected to minimize potential biases in restorative perception during video viewing, as design majors might possess professional knowledge or critical perspectives that could influence their responses to the visual stimuli. The analysis using G*Power 3.1.9.7 indicates that a total sample size of 45 participants is required to achieve a statistical power of 80% with a small to medium effect size of 0.15, a significance level (α) of 0.05, and a correlation among repeated measures of 0.5. Considering potential dropouts and other issues (e.g., signal disturbance), we recruited 60 participants for the experiment and ultimately collected data from 55 individuals (30 males and 25 females).

### 2.3 Outcome measures

The experiment mainly evaluates the restorative outcomes of the different greenway settings through three aspects: Participants’ attentional level changes before and after watching the mixed greenway video, participants’ brainwave responses during the watching process, and their valuation of the restorative quality of the different greenway types.

#### 2.3.1 Attentional level test

We assessed attention level of the subjects using the Necker Cube Pattern Control Test (NCPCT). The Necker Cube is a wire-frame cube, which can be seen in two different orientations. The frequency at which the cube appears to flip or change perspectives is the key factor in measuring attention (Hulburt, 2011). A lower score on the test signifies a higher level of sustained attention, while a higher score indicates greater attention fatigue. The NCPCT has proven to be a reliable measure of attention fatigue in restorativeness studies (Hartig et al., 2003; Tennessen and Cimprich, 1995). We chose the NCPCT as the attentional test because it can be displayed on screen, is easy to operate, and time-efficient. While during the experimental process, a few participants reported being unable or have difficulties in observing the rotation of the Necker Cube, despite being provided with instructions on how to observe it. Ultimately, 39 out of the 55 participants completed the Necker Cube task.

#### 2.3.2 Electroencephalography monitoring

Electroencephalographic activity was measured with a 14-channel Epoc X EEG headset from Emotiv. This device provides a sampling rate of up to 256 samples per second and an internal rate of 2,048 Hz, with a resolution of 14 bits. It utilizes a proprietary 2.4 GHz wireless connection for real-time data transmission. The sensors, made of silver/silver chloride (Ag/AgCl) with felt and saline, ensure reliable signal conduction and adhere to the 10–20 system placement. Electrodes are placed at specific points identified by letters which indicate underlying brain regions (F = Frontal, T = Temporal, P = Parietal, O = Occipital) and numbers (odd for the left hemisphere, even for the right, z for midline) (Emotiv, n.d.-a). The EPOC X device uses P3/P4 referencing, with P3 serving as the reference electrode for brainwave signals and P4 used for noise cancelation (Emotiv, n.d.-b). A total of 55 participants completed the EEG data collection, with 52 providing qualified EEG signals that can be processed further.

#### 2.3.3 Valuation of the restorative quality

The short version of the Perceived Restorativeness Scale consisted of five items was used in this study, which was developed by Berto (2005) and includes one item for each factor from the original 16-item scale created by Korpela and Hartig (1996). Simplifying the measurement makes it more practical for experiments by saving time and reducing subject fatigue. The statements in the PRS short version are rated on a 7-point scale, where 1 signifies “not at all” and 7 denotes “very much.” All 55 participants completed the PRS scale without any missing data. The internal consistency (Cronbach’s alpha) was 0.866.

### 2.4 The procedure

The day before the experiment, subjects were notified that the entire session would last about 1 h, and were also reminded to rest well before the experiment, avoiding late nights, coffee, alcohol, and other stimulating substances. Additionally, an introduction to the NCPCT was provided to familiarize subjects with the content of the test.

**FIGURE 1:**
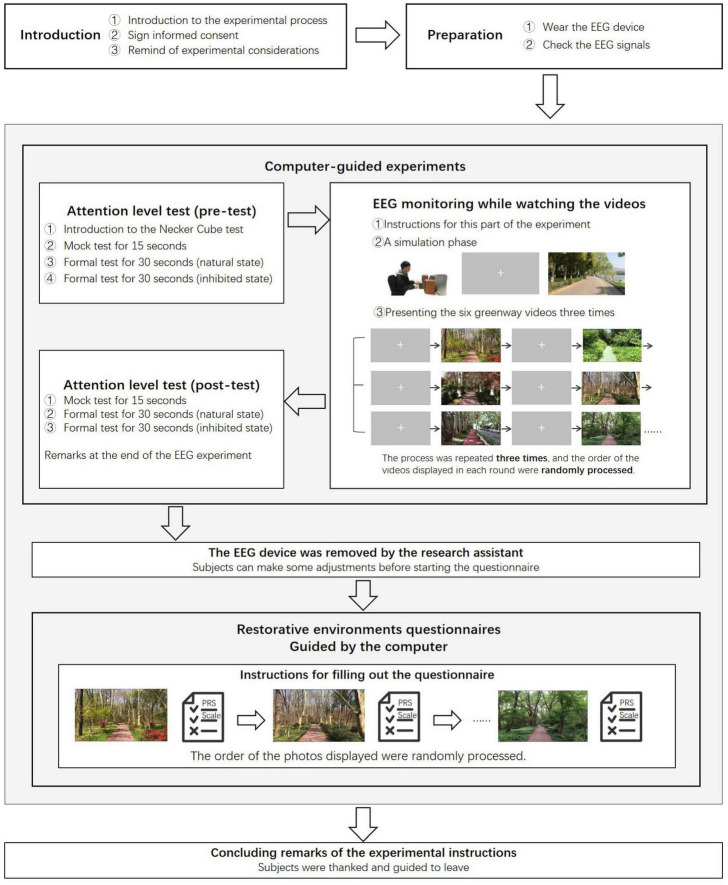
Experiment procedure.

The experiments were conducted between 6:30 and 9:00 p.m., a time when most participants having completed a full day of studies and in need of attentional restoration. After the subjects arrived at the laboratory, they were fitted with EEG equipment with their consent, and adjustments were made to ensure that the device connections and data transmission were functioning properly. The participants then adjusted to a comfortable sitting position, and the experimenter initiated the experimental program. Subjects then completed the experimental process following the guidance displayed on the computer screen. The experimental program had been designed in advance. On the first page, it presented the testing phases of the experiment, advising subjects to minimize body and head movements while wearing the EEG equipment, and asked them to imagine being immersed in the setting when viewing the environmental videos. Once ready and after providing informed consent, participants could press the space bar to begin the actual experiment (Figure 1).

The initial phase of the program involved using a Necker cube to test attention. After a 15-s simulation test, the subjects were asked to observe the cube and count the number of perspective shifts during two 30-s intervals: The first was conducted naturally, and the second in a controlled manner, where they were instructed to maintain a single perspective to inhibit involuntary reversals. The second phase involved watching videos of greenway settings. The process began with a 15-s simulation phase, during which participants viewed a scene of a greenway through an urban park of average quality, which was unrelated to any urban forest greenway scenes. This was followed by the formal test phase, which included six 30-s videos from urban forest greenways, each played three times, for a total of 90 s per video. This approach aimed to reduce viewer fatigue and enhance data reliability by minimizing cognitive load and providing slightly longer, stable exposure. The order of video playback was randomized to avoid the influence of sequence. Before each video of the greenways, a 6-s blank screen with a fixation point (a plus sign) in the center was displayed for a break. The total playback duration was approximately 10 min, during which the EEG monitoring device continuously recorded the subjects’ brain activity. The Necker cube test was conducted again after the video watching, and then the experimenter removed the EEG equipment for the subjects.

Then the experimenter provided the subjects with a paper-based questionnaire, which they used to evaluate the restorative quality of the greenway scenes they had observed. Screenshots from the video were displayed on the screen (Table 1), and all participants viewed the same set of photos (one for each video, totaling six), though in a random sequence. Subjects were instructed to assess the images based on their viewing experience, note the photo number, and then complete the questionnaire. After evaluating one photo, they could press the space key to proceed to the next photo. Participants rated the perceived restorativeness of the scenes after the video watching to avoid interference during EEG measurements, as they needed to maintain their posture. This also helped control EEG recording time, to prevent the saline from drying and affect data quality. This method allowed for a presentation of the photos from the videos, and gave the subjects the flexibility to fill out the questionnaires at their own pace. The questionnaires were designed to assess the restorative quality of each greenway scene, rather than their immediate restoration outcomes after video viewing, which suggests that the possibility of memory consolidation or fading may be limited. Although the questionnaire did not explicitly assess participants’ familiarity with the scenes depicted in the videos, post-experiment verbal inquiries revealed that most participants had not visited any of the locations, suggesting that participants were generally unfamiliar with the scenes shown. After completing the experiment, the experimenter thanked the subjects and guided them out.

### 2.5 Data analysis

EEG data was processed using EEGLAB version 2022.1. The collected data was filtered with a band-pass filter from 1 to 40 Hz, and a notch filter was applied between 48 and 52 Hz. Values above 100 μV were considered artifacts due to the low amplitude of the EEG signals. Artifacts were subsequently removed using Adjust 1.1 when appropriate. Following this, we extracted three pieces of 30-s segments of EEG data corresponding to each of the six greenway videos (played three times) using pre-set markers. The extracted data was then segmented into 2-s epochs, a method suitable for static EEG data analysis. The data were then manually reviewed, with each epoch thoroughly examined for potential artifacts such as excessive noise, abnormal amplitude fluctuations, and movement-related disturbances. Any epochs containing these artifacts were rejected, with the process conducted by a trained EEG research assistant who followed standardized protocols to ensure consistency. Additionally, any subject data with fewer than 50% valid epochs were excluded to ensure robust and reliable analysis. This process resulted in an average of 38 valid epochs per greenway scene per person, with data nested within 40 individuals.

Using the processed files, study sets were created for further analysis. The EEG signals were categorized into five frequency bands: Theta waves (4–7 Hz), alpha waves (8–12 Hz), low beta waves (12–15 Hz), mid-range beta waves (15–20 Hz), and high beta waves (18–40 Hz). Theta waves typically associate with deep relaxation, meditation, and sleep. Alpha waves are prominent during a normal wakeful state of quiet rest, which can be linked to relaxation and a reduced level of mental activity, reflecting calm and restoration. Beta waves are present when a person is alert and actively thinking, with high beta waves particularly associated with significant stress, anxiety, paranoia, high energy, and heightened arousal (Abhang, et al., 2016). Spectrogram analysis was conducted to examine differences in various rhythmic brain activities while watching the greenway videos, using the LIMO (Hierarchical Linear Modeling) EEG toolbox, to compare different types of greenways and the tended greenway across four seasons. LIMO allows for testing each time frame and electrode of the EEG array and can control for Type I errors using clustering correction, and it can handle both within-subject variance (single trial analyses) and between-subject variance. The main effect of gender and its interactions with greenway subtypes were insignificant after Bonferroni corrections and were not considered in the following analysis.

Shapiro-Wilk test and visual methods like histograms or Q-Q plots were used to assess normality. Due to non-normality of the data in attentional level test, Wilcoxon Signed Ranks tests were conducted to analyze the differences in the number of flips in the NCPCT before and after watching greenway videos, in both natural and inhabited states, reflecting changes in the subjects’ attention levels. In addition to the pre-post difference test conducted for the same state, we also found that the difference between the pre-test (inhibited state) and post-test (natural state) scores was insignificant (*Z* = –1.686; *p* = 0.092), suggesting that the practice effects of the NCPCT are limited.

Multilevel modeling was employed to explore the differences in perceived restorativeness among six greenway settings, varying by type and season, as each subject evaluated all scenarios. Repeated measurements (level 1) were nested within individuals (level 2). Types of greenway settings were treated as fixed effects, using the winter tended greenway scene as a reference. The significance level was set at *p* < 0.05. Statistical analyses were conducted using R version 4.1.1.

## 3 Results

### 3.1 Testing for attention level before and after video watching

The changes in the attentional level of the subjects before and after watching greenway videos were assessed using the NCPCT (*N* = 39). In the natural state, where subjects counted the reversals of the cube’s flips freely, the average number of flips in 30 s was 7.79 (*SD* = 3.65) before watching the videos, which reduced to 5.95 (*SD* = 2.99) after watching. Wilcoxon Signed Ranks test revealed that this reduction was significant (*Z* = –4.25, *p* < 0.001), indicating that watching urban forest greenway videos can partially restore attentional levels when subjects were in natural state. In the inhibited state, where subjects were asked to maintain a single perspective of the cube, the average frequency of flips was 5.21 (*SD* = 3.28) before and 4.56 (*SD* = 2.48) after watching the videos. A decrease in the number of flips was also observed in the inhibited state, and the change was statistically significant (*Z* = –2.12, *p* = 0.034), indicating that urban forest greenway scenes help subjects focus their attention when needed (Table 2). The above results confirm the Hypothesis 1.

**TABLE 2:** Wilcoxon Signed Ranks test results for the changes in number of Necker Cube reversals before and after watching videotaped urban forest greenway scenes.

Mode of state	Post-test-Pre-test	N	Mean rank	Sum of ranks	Z	P
Natural state	Negative ranks	30	21.02	630.50	–4.25	< 0.001
	Positive ranks	7	10.36	72.50		
	Ties	2				
Inhibited state	Negative ranks	22	14.25	313.50	–2.12	0.034
	Positive ranks	7	17.36	121.50		
	Ties	10				

### 3.2 Effects of different urban forest greenway videos on brain activity

This study collected qualified EEG data from 52 subjects. Following preprocessing, epoch extraction, and manually review, data from 40 subjects were used in the formal analysis. Firstly, this study analyzed the power spectral intensity across different subtypes of urban forest greenways, including tended greenways, urban greenways, and wilderness greenways, with videos recorded in the same summer season (Table 3).

**TABLE 3:** Means [Log Power 10 * log10(μV^2^)] and standard deviations for EEG waves (O1: Left occipital lobe, O2: Right occipital lobe according to International 10–20 system) in response to subtypes of urban forest greenways in summer.

Wave length	Lead	Tended greenway	Urban greenway	Wilderness greenway
Theta waves (4–7 Hz)	O1	46.95 (0.63)	47.03 (0.56)	47.03 (0.73)
	O2	49.08 (0.71)	48.96 (0.73)	49.11 (0.69)
Alpha waves (8–12 Hz)	O1	46.24 (0.26)	45.87 (0.16)	46.04 (0.19)
	O2	48.63 (0.51)	48.36 (0.34)	48.52 (0.40)
Low beta waves (12–15 Hz)	O1	45.18 (0.55)	44.91 (0.64)	45.05 (0.73)
	O2	47.67 (1.03)	47.59 (0.95)	47.75 (0.84)
Mid-range beta waves (15–20 Hz)	O1	42.87 (0.53)	42.83 (0.56)	42.91 (0.52)
	O2	44.92 (0.59)	44.88 (0.60)	44.99 (0.65)
High beta waves (18–40 Hz)	O1	41.78 (0.62)	41.82 (0.58)	41.75 (0.64)
	O2	43.34 (0.86)	43.36 (0.76)	43.30 (0.87)

**FIGURE 2:**
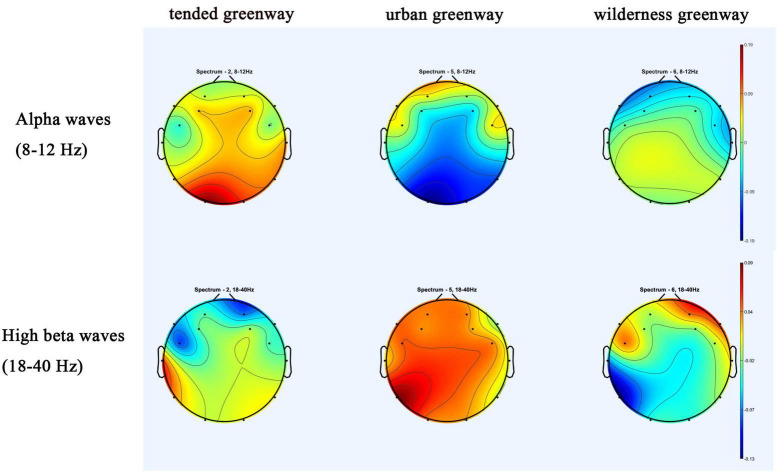
Topographic map of the EEG wave frequency distribution for all subjects, in response to the three subtypes of urban forest greenways, obtained by subtracting the individual subject mean spectrum and plotting the averaged topography over the frequency range.

Figure 2 shows topographic maps of EEG amplitudes for all subjects across the three subtypes of urban forest greenways at alpha and high beta waves. Red indicates positive waves, and blue indicates negative waves. The closer the color is to red or orange, the higher the intensity of the corresponding brainwaves, while colors closer to blue or green, the lower the intensity. The topographic maps showed that, compared to the urban greenway, watching scenes of tended and wilderness greenways was associated with higher alpha waves and lower high beta waves, and suggest a greater potential for attention restoration.

**FIGURE 3:**
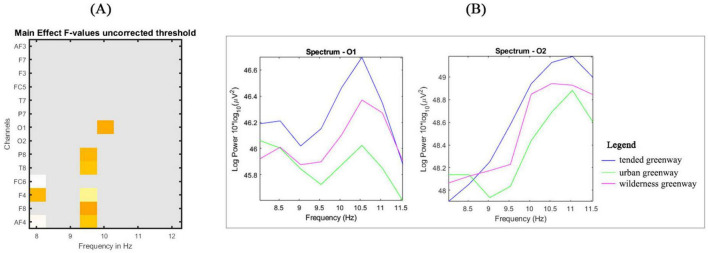
Spectrogram analysis of alpha waves (8– 12 Hz) for different subtypes of urban forest greenways (tended greenway, urban greenway, and wilderness greenway) in the raw comparisons: **(A)** main effects *F*-values masked for statistical significance (*p* < 0.05); **(B)** spectrum plots in both channels O1 and O2.

Using LIMO EEG, statistical comparisons were conducted to identify group differences. As shown in Figure 3, the main effect F-values across different frequencies highlight significant differences around the 10 Hz frequency in channels such as O1, P8, T8, F8, and AF4 (*p* < 0.05) in the raw comparisons without multiple comparison correction. Power spectral density plots for the O1 and O2 electrodes showed the highest alpha wave activity in the tended greenway, followed by the wilderness and urban greenways. However, after applying clustering correction, the biggest observed cluster mass (14.81) was lower than the threshold for significant cluster mass (68.82), indicating that the effect was not significant after correction, which runs counter to Hypothesis 2.

Secondly, this study analyzed the power spectral intensity of the tended greenway across the four seasons—spring, summer, autumn, and winter. The mean and standard deviation values for each season’s greenway across different bands were calculated (Table 4). However, using Limo EEG calculations, the differences between the seasons did not show clear significance. Therefore, Hypothesis 3 was not supported by the results.

**TABLE 4:** Means [Log Power 10 * log10(μV^2^)] and standard deviations for EEG waves in response to tended greenway scenes of different seasons.

Wave length	Lead	Spring scene	Summer scene	Autumn scene	Winter scene
Theta waves (4–7 Hz)	O1	47.05 (0.65)	46.95 (0.63)	46.99 (0.66)	47.05 (0.52)
	O2	49.02 (0.66)	49.08 (0.71)	48.98 (0.77)	49.08 (0.55)
Alpha waves (8–12 Hz)	O1	46.12 (0.20)	46.24 (0.26)	46.29 (0.27)	46.33 (0.21)
	O2	48.47 (0.29)	48.63 (0.51)	48.69 (0.49)	48.74 (0.41)
Low beta waves (12–15 Hz)	O1	44.94 (0.52)	45.18 (0.55)	45.13 (0.54)	45.01 (0.61)
	O2	47.64 (0.89)	47.67 (1.03)	47.95 (0.77)	47.80 (0.95)
Mid-range beta waves (15–20 Hz)	O1	42.89 (0.54)	42.87 (0.53)	42.94 (0.60)	43.09 (0.57)
	O2	44.90 (0.53)	44.92 (0.59)	45.09 (0.60)	45.11 (0.61)
High beta waves (18–40 Hz)	O1	41.73 (0.64)	41.78 (0.62)	41.77 (0.61)	41.96 (0.64)
	O2	43.18 (0.88)	43.34 (0.86)	43.29 (0.88)	43.36 (0.83)

### 3.3 Assessing the perceived restorativeness of different urban forest greenways

Descriptive statistics indicated that subjects (*N* = 55) rated the restorative quality of the tended greenway highest, with mean scores as follows: Spring (*M* = 5.59, *SD* = 0.79), summer (*M* = 5.40, *SD* = 0.87), autumn (*M* = 5.15, *SD* = 1.23), and winter (*M* = 4.61, *SD* = 1.18). The wilderness greenway in summer (*M* = 4.72, *SD* = 1.27) had a similar perceived restorativeness score to the tended greenway in winter. The urban greenway in summer received the lowest score (*M* = 3.18, *SD* = 1.21) (Table 5).

**TABLE 5:** Means and standard deviations for overall perceived restorativeness scale (PRS) value of different greenway types in different seasons.

Type	Season	Mean	S.D.
Tended greenway	Spring scene	5.59	0.79
	Summer scene	5.40	0.87
	Autumn scene	5.15	1.23
	Winter scene	4.61	1.18
Urban greenway	Summer scene	3.18	1.21
Wilderness greenway	Summer scene	4.37	1.27
Total		4.72	1.36

Multilevel modeling, incorporating individual effects, was used to analyze subjects’ valuations of perceived restorativeness for different types of greenways across seasons. A Shapiro-Wilk test for normality indicated that the residuals were normally distributed, *W* = 0.99, *p* = 0.190, suggesting that the data are appropriate for analysis using a mixed model. Compared to the tended greenway in winter, the urban greenway in summer was rated significantly lower in perceived restorativeness (*B* = –1.43, *t* = –8.11, 95% CI: –1.77 to –1.09), indicating that tended greenways generally offer better restorative effects than urban greenways, which was in line with H4a. When using the wilderness greenway in summer as a reference for comparison, the tended greenway showed better restorative effects (*B* = 1.02, *t* = 5.80, 95% CI: 0.68–1.37), whereas the urban greenway demonstrated lower restorative effects (*B* = –1.19, *t* = –6.77, 95% CI: –1.54 to –0.85).

Among the four seasons, the tended greenway exhibited varying restorative qualities, suggesting seasonal influences. Compared to winter, the tended greenway received higher ratings in other seasons, particularly in spring (*B* = 0.98, *t* = 5.57, 95% CI: 0.64–1.33), followed by summer (*B* = 0.79, *t* = 4.46, 95% CI: 0.44–1.13), and autumn (*B* = 0.53, *t* = 3.03, 95% CI: 0.19–0.88) (Table 6). The above results confirmed the Hypothesis 4b.

**TABLE 6:** Evaluation of overall Perceived Restorativeness Scale (PRS) for different greenway types using multilevel modeling.

Variable	B	SE	*T*-value	95% CI	
				Lower	Upper
**Fixed effects**					
(Intercept)	4.61***	0.15	30.90	4.32	4.90
Tended greenway (Spring) ^1^	0.98***	0.18	5.57	0.64	1.33
Tended greenway (Summer) ^1^	0.79***	0.18	4.46	0.44	1.13
Tended greenway (Autumn) ^1^	0.53**	0.18	3.03	0.19	0.88
Urban greenway (Summer) ^1^	–1.43***	0.18	–8.11	–1.77	–1.09
Wilderness greenway (Summer) ^1^	–0.24	0.18	–1.34	–0.58	0.11
**Random effects**					
Individual	0.60 (0.92)				

^1^The greenway scenes were referenced to the scene of “Tended greenway (Winter).”

**p < 0.01,

***p < 0.001.

## 4 Discussion

### 4.1 Greenway scenes for attention restoration

The results of the study indicate that a 10-min video of mixed urban forest greenways is sufficient to achieve attention restoration. The video scenes presented a dynamic, linear stroll along the greenway, enhancing the sense of walking experience. This type of video proved effective in restoring attention. However, due to the limitations of the experimental design and arrangement, a detailed exploration of attention restoration in response to videos featuring individual subtypes of urban forest greenways has not been conducted.

### 4.2 Different subtypes of urban forest greenways

This study considers the planning and management of urban forest greenways, to provide useful information for enhancing their restorative quality. Results from the perceived restorativeness suggested that, compared to urban greenways running closely parallel with city roads, tended greenways and wilderness greenways, with greenery along both sides of the trails, offer better opportunities for restoration. These results are primarily derived from subjective restorative assessments, as no significant differences were observed between the subtypes of urban forest groups in the EEG data. The differences in significance levels between the EEG data and perceived restorative assessments may be attributed to the differing focuses of the two assessments: EEG measures the participant’s immediate brain activity while watching the video, while PRS captures more cognitive and affective evaluations, focusing on evaluating the restorative potential of the scene as perceived by the subjects. Additionally, although the 14-channel Epoc X EEG headset can collect reliable signals with advantages of convenience and comfort, it may have lower spatial resolution and be more susceptible to fluctuations and noise compared to EEG devices with more channels. The reduction in sample size after data preprocessing may also contribute to the differences.

This finding aligns with previous research (Sullivan and Chang, 2011; van den Berg et al., 2007), suggesting that having greenways directly adjacent to city roads with only a simple shrub barrier is less than ideal. Such a design may, as it reduces visible greenery and increases disturbances from vehicles and noise (Zhong et al., 2023). This provides insights for selecting greenway routes. While it is common for greenways around mountains to be near city roads, leaving a green buffer of at least 10 m on the city side with multi-layered planting can notably reduce the negative impact and enhance the user experience (Paudel and States, 2023). Some may argue that reserving buffer zones for greenway routes could increase costs. However, our experiments show that the benefits of careful route selection are significant, often outweighing the choice of planting methods. For example, our study demonstrates that the wilderness greenway—a low-cost option—provides better restorative benefits than the urban greenway. This highlights the need to evaluate all economic factors when selecting a greenway route.

In addition to route selection, it is also important to consider the design of the vegetation on both sides of the urban forest greenway. This study found that, compared to the wilderness greenway, the tended greenway showed better restorative effects, indicating that carefully designed and managed green spaces can enhance the restorative benefits for visitors. This may be because tended greenways provide a sense of security and are aesthetically pleasing, as confirmed by previous literature (van den Berg et al., 2014). A sense of security can enhance perceived restorativeness, and a preference for beauty is closely related to restorative perception (Twedt et al., 2019). It is interesting to note that in the stimuli materials, the greenery visibility rate for both the tended greenway and the wilderness greenway is very similar, with only the path itself visible as the hardscape. The differing restorative effects between these two types of greenways highlight the importance of landscape quality; simply having greenery is not enough.

Landscape quality is a complex concept, but here we offer a few perspectives. For instance, the structural diversity of planting can enhance the sense of “being away,” providing a contrast to urban landscapes and increase the sense of “soft fascination.” However, it is important to consider compatibility in design, as excessive layering may create a sense of confusion, so well-maintained urban forest greenways tend to be more restorative, as they exhibit the characteristic of “compatibility.” Other factors, such as perceived openness, trail width and material, and signs of maintenance, may also influence the restorative experience and deserve further investigation. In the video scenes used in our study, while the tended greenway already offers strong restorative benefits, there is potential for further enhancement. By incorporating some of the advantages of the wilderness greenway, such as denser vegetation that effectively blocks noise and enriches the soundscape with more birdsong, the tended greenway could provide a stronger sense of “being away” and “soft fascination.” This would create a more immersive environment while still preserving the managed appearance and sense of security that contribute to its appeal.

### 4.3 Seasonal changes of tended greenways

The tended greenway tested in this experiment is typical in design, reflecting the climate of subtropical regions in the eastern parts of continents, characterized by distinct seasons with hot summers and relatively cold but snowless winters. By evaluating the restorative potential of the tended greenway in four seasons, this study provides a new perspective on greenway design, which can help promote the restorative effects of greenways for walkers year-round and allow for better utilization. This study can help in understanding how to create different vegetation layers and select appropriate plant species, such as evergreens, deciduous trees, or colorful foliage trees.

Tall deciduous trees along greenways provide shade in the summer and allow sunlight to penetrate in the winter after the leaves have fallen. This design is crucial for maintaining a comfortable temperature and enabling people to enjoy the sunshine during colder months. However, the reduction in visible greenery during winter can affect the natural feel of the greenway and diminish its restorative effects. To address both climate adaptation and the enhancement of restorativeness, vegetation should be strategically planned based on human scale. Light-blocking vegetation layers should be placed above eye level, with deciduous trees serving this purpose (Mooney, 2019). Below eye level, evergreen plantings can be added to enhance the restorative quality of the greenway landscape in winter, with these plants set slightly back from the greenway edge to avoid blocking sunlight (Robinson, 2016).

The study also indicates that the restorative quality of greenway landscapes is not solely dependent on the amount of greenery in view. Interestingly, participants rated their restorative experience higher in spring than in summer, possibly because the vibrant red new leaves and tender yellow-green hues of early spring provide a soft fascination through subtle color gradations. Nature provides a rich palette, and incorporating diverse plant colors and textures into the design can enhance the restorative potential of greenways. This aligns with previous research showing that non-green plants can also be restorative (Bielinis et al., 2021; Chen et al., 2023), but contrasts with studies suggesting that autumn and winter scenes may be more effective in reducing fatigue than those in spring and summer (Litleskare and Calogiuri, 2023). The effectiveness of seasonal scenes in promoting restoration likely depends on local climate, as well as cultural and social factors, and may require further investigation.

When considering the need for regular mental and physical restoration throughout the year, as repeated exposure to natural environments can lead to cumulative benefits for both mental and physical health (Dzhambov et al., 2019; Jones et al., 2021), it’s important to recognize that the restorative benefits of urban forest greenways vary with the seasons. The level of rejuvenation experienced during daily walks may change depending on the time of year. In winter, when the greenway’s restorative effects are naturally diminished, it may be beneficial to extend the duration of the activity or to explore other environments that offer restorative benefits to maintain overall well-being.

### 4.4 Limitations and future research

In this study, a few factors affect the final sample size. Given that some participants may have had difficulties with the Necker Cube task, their decision to withdraw from this portion of the test was respected. This withdrawal resulted in a slightly lower sample size, potentially reducing the statistical power of the analysis. Although the dropout rate for the Necker Cube task in similar age groups has not been widely reported, one study addressed data handling by excluding participants who exhibited fewer than five reversals within one min, according to its research objectives, resulting in approximately 71% validity of the collected data (Wilson et al., 2023). This suggests that while the Necker Cube is a highly effective tool for measuring attention, it may not be equally well accepted by all participants. Future studies could also consider other attentional tests (e.g., SART, Sustained Attention to Response Task) and other established paradigm, which may yield more reliable and generalizable results. As for EEG recording, during data collection, a few participants had their data discarded due to poor signal quality, and several additional samples were lost during the data cleaning and noise removal process. This also impacted the sample size, which may have influenced the robustness of the analysis.

Considering the limited sample size, a repeated measures design was adopted in this study, allowing each participant to experience multiple conditions. While this approach increased within-subject efficiency, it did not permit comparisons between different subtypes in terms of their impact on attentional levels. To further control the duration of the experiment and reduce participant fatigue, the analysis of seasonal effects focused exclusively on tended greenways. In addition, due to recruitment constraints, the sample consisted solely of university students aged 20–25, which limits the generalizability of the findings. Future research could address these limitations by recruiting a larger and more diverse participant pool to examine the restorative effects of different seasons across various subtypes of urban forest greenways, while also allowing for improved consideration of individual differences such as gender and personality traits. Furthermore, a between-subjects design could be employed to explore how different subtypes of urban forest greenways influence attention following video exposure, enabling comparisons between groups.

Another limitation of this study is that all six greenway videos represented urban forest greenways, without the inclusion of a non-restorative or urban baseline environment for comparison. This lack of contrast makes it more difficult to isolate the specific restorative effects of urban forest greenways. As a result, the EEG data, while showing trends consistent with the hypothesis, did not reach statistically significant differences. Future studies could include a control condition, such as a typical urban environment (e.g., walking along a road), to provide a clearer comparison and assess the restorative effects of urban forest greenways.

Conducting experiments in a controlled laboratory setting offers strong experimental control by minimizing external distractions. But this approach may not fully replicate the real-world experience of the settings, particularly in terms of seasonal sensations such as temperature, humidity, and ambient sounds (Conniff and Craig, 2016). These sensory dimensions are difficult to simulate through video, which may limit ecological validity. Future research could consider conducting *in situ* experiments in real outdoor settings to capture a more immersive and realistic restorative experience.

## 5 Conclusion

As a vital trail for experiencing the urban forest, greenways provide citizens with opportunities to connect with nature and engage in physical activities. This study suggest that different subtypes of urban forest greenways may have different effects on restorativeness, which are primarily reflected in objective measures. Specifically, the preliminary findings indicate that the tended greenway might have more restorative effects than the wilderness and the urban greenway, providing insights for route selection and landscape management to enhance restorative benefits. The study also explored the impact of seasonal factors on the restorative benefits of greenways and found that tended greenways in other seasons, particularly spring and summer, may be perceived more restorative than in the winter. This can assist users in making informed choices for a restorative experience along the urban forest greenway. It can also offer ideas for designing vegetation landscapes along the greenway to provide year-round restorative benefits, such as by effectively combining deciduous trees and evergreens at different levels.

## Data Availability

The raw data supporting the conclusions of this article will be made available by the authors, without undue reservation.
